# House screening with insecticide-treated netting provides sustained reductions in domestic populations of *Aedes aegypti* in Merida, Mexico

**DOI:** 10.1371/journal.pntd.0006283

**Published:** 2018-03-15

**Authors:** Azael Che-Mendoza, Anuar Medina-Barreiro, Edgar Koyoc-Cardeña, Valentín Uc-Puc, Yamili Contreras-Perera, Josué Herrera-Bojórquez, Felipe Dzul-Manzanilla, Fabian Correa-Morales, Hilary Ranson, Audrey Lenhart, Philip J. McCall, Axel Kroeger, Gonzalo Vazquez-Prokopec, Pablo Manrique-Saide

**Affiliations:** 1 Vector Biology Department, Liverpool School of Tropical Medicine, Liverpool, United Kingdom; 2 Unidad Colaborativa para Bioensayos Entomologicos, Universidad Autonoma de Yucatan, Merida, Yucatan, Mexico; 3 Centro Nacional de Programas Preventivos y Control de Enfermedades, Secretaria de Salud, Ciudad de Mexico, Mexico; 4 Centers for Disease Control and Prevention, Entomology Branch, Atlanta, Georgia, United States of America; 5 Special Programme for Research and Training in Tropical Diseases (TDR), World Health Organization, Geneva, Switzerland; 6 Department of Environmental Sciences, Emory University, Atlanta, Georgia, United States of America; North Carolina State University, UNITED STATES

## Abstract

**Background:**

There is a need for effective methods to control *Aedes aegypti* and prevent the transmission of dengue, chikungunya, yellow fever and Zika viruses. Insecticide treated screening (ITS) is a promising approach, particularly as it targets adult mosquitoes to reduce human-mosquito contact.

**Methodology/Principal findings:**

A cluster-randomised controlled trial evaluated the entomological efficacy of ITS based intervention, which consisted of the installation of pyrethroid-impregnated long-lasting insecticide-treated netting material fixed as framed screens on external doors and windows. A total of 10 treatment and 10 control clusters (100 houses/cluster) were distributed throughout the city of Merida, Mexico. Cross-sectional entomological surveys quantified indoor adult mosquito infestation at baseline (pre-intervention) and throughout four post-intervention (PI) surveys spaced at 6-month intervals corresponding to dry/rainy seasons over two years (2012–2014). A total of 844 households from intervention clusters (86% coverage) were protected with ITS at the start of the trial. Significant reductions in the indoor presence and abundance of *Ae*. *aegypti* adults (OR = 0.48 and IRR = 0.45, P<0.05 respectively) and the indoor presence and abundance of *Ae*. *aegypti* female mosquitoes (OR = 0.47 and IRR = 0.44, P<0.05 respectively) were detected in intervention clusters compared to controls. This high level of protective effect was sustained for up to 24 months PI. Insecticidal activity of the ITS material declined with time, with *~*70% mortality being demonstrated in susceptible mosquito cohorts up to 24 months after installation.

**Conclusions/Significance:**

The strong and sustained entomological impact observed in this study demonstrates the potential of house screening as a feasible, alternative approach to a sustained long-term impact on household infestations of *Ae*. *aegypti*. Larger trials quantifying the effectiveness of ITS on epidemiological endpoints are warranted and therefore recommended.

## Author summary

An ITS based intervention, screened houses with insecticide-treated netting (long-lasting insecticidal nets permanently fixed with aluminium frames on external doors and windows), in Merida city, Mexico. Screened houses had >50% less chance of having *Ae*. *aegypti* mosquitoes indoors and importantly, >50% fewer female *Ae*. *aegypti* in comparison with non-screened houses (control houses). The impact was sustained over 2 years. House screening could provide a feasible sustainable method to reduce human-vector contact inside houses and could potentially be scaled up to protect against mosquito-borne diseases, particularly in locations where the simultaneous transmission, or threat, of dengue, chikungunya, yellow fever and Zika is occurring.

## Introduction

The development of effective and long-lasting methods for the control of the mosquito *Aedes aegypti* is a top global health priority, especially in the current epidemiological context of multiple co-circulating *Aedes*-borne diseases (dengue, yellow fever, chikungunya, Zika, mayaro) [1,2]. Vector control programs strongly rely on insecticide-based approaches such as house-to-house hand-application of larvicides to water holding containers, ultra-low volume adulticiding and thermal fogging [3]. Previous research suggests that such methods of vector control, particularly when applied in isolation, have little, if any, impact on controlling adult mosquito populations or preventing disease [2,4–7]. This is largely because their effect is transient and depends on continued re-application to achieve a measurable entomological impact [8]. The emergence of insecticide resistance in *Ae*. *aegypti* and the challenges represented by highly variable resistance patterns over space and time [9] further limits the effectiveness of insecticide-only traditional strategies and provides a strong justification for the development of integrated vector management plans for *Aedes-*borne disease control [10]. Achieving a measurable impact on *Aedes*-borne diseases requires a re-formulation of current vector control strategies and a renewed focus on both lowering adult vector abundance and preventing human-vector contact [1].

When used properly, long-lasting insecticide treated netting (LLIN), most commonly used as bednets, have been shown to provide an effective way to reduce human-vector contact from multiple pathogens transmitted by endophilic night-biting mosquitoes [11–15]. The deployment of LLIN material as window curtains (insecticide treated curtains, ITCs) has proven to reduce indoor *Ae*.*aegypti* densities and theoretically reduce dengue transmission risk [16–20]. While ITCs can be easily implemented within *Ae*. *aegypti*-endemic areas, recent studies have revealed challenges related to their handling and sustained usage. For example, in Iquitos, Peru, a sociological study found that correct use of ITCs fell dramatically over time (45% in the second year of deploying) [21]. In addition, the efficacy of ITCs can be compromised when curtains remain open during daytime or when all house entry points cannot be protected [18–20,22,23]. ITCs were shown to be most effective when houses had fewer and smaller windows and doors and where coverage of the intervention was particularly high [2].

Modifying houses to make them refractory to vector entrance is gaining renewed momentum as a paradigm for mosquito control [24–28]. As most human exposure to *Ae*. *aegypti* occurs indoors [29], the use of screens as physical barriers to the entrance of mosquitoes has been found as a protective housing feature in many observational epidemiological studies [30–33]. Housing improvement as a public health intervention is being considered for *Ae*. *aegypti* control in Mexico as part of an integrated vector management strategy [27]. A housing improvement based intervention called was developed in Mexico to evaluate the entomological impact of applying insecticide treated screening (ITS) with LLIN material permanently to doors and windows [34,35]. Cluster-randomised controlled trial (RCT) performed in the city of Acapulco, showed that ITS had an immediate but also a sustained (~2yr) impact on indoor-adult *Ae*. *aegypti* infestations, even in the presence of high pyrethroid resistance in the local *Aedes* population [34,35]. Such results suggest that ITS could act both as a physical and a chemical barrier to mosquitoes. ITS was viewed positively by the community, with a perceived efficacy on mosquito abundance and biting, and a perceived reduction in other domestic pests [36].

Because of the co-endemicity risk of dengue (DEN), chikungunya (CHIK) and Zika (ZIK) viruses in a large proportion of *Ae*. *aegypti* infested cities of Mexico, the National Ministry of Health pursued the potential of house screening for disease prevention [37]. The promising results in Acapulco led to a follow-up initiative, applying the intervention to a new metropolitan area to generate further evidence of the efficacy and limitations of the strategy. Here, we report results of a RCT evaluating the entomological impact of the ITS intervention on indoor *Ae*. *aegypti* populations in the city of Merida, Mexico.

## Methods

### Study site

The study took place in the city of Merida, located in the State of Yucatan, Mexico. Merida is the state capital and the major urban center with a population of 814,435 inhabitants living in 272,418 households [38]. Dengue is highly prevalent in Merida. More than 50% of all dengue cases reported in Yucatan State occurred in Merida. During 2005–2015 dengue case reports in the city ranged between 437 and 3,953, and incidence rates (per 100,000 people) ranged between 49 and 254. Fatalities caused by dengue have been negligible except for 51 deaths reported during 2011–2013, with a lethality less than 1%. In the national context, Merida is one of three Mexican cities (above of 800,000 inhabitants) that have reported the highest proportion of dengue cases in the last 15 years (3.7%), together with Acapulco (3.1%) and Veracruz (2.4%) [39]. The first cases of chikungunya and a subsequent outbreak (1,531 cases) occurred in 2015 and transmission is ongoing [39]. Zika transmission was detected in May 2016 and cases continue to be reported [39]. Previous studies in Merida on productive container types for *Ae*. *aegypti* immatures have incriminated disposable containers and buckets/pots, and other rain-filled objects left in backyards [40–42]. Non-residential habitats, such as subsurface catch basins, have been also identified as productive *Ae*. *aegypti* habitats [43,44].

### Study design

**Fig 1:**
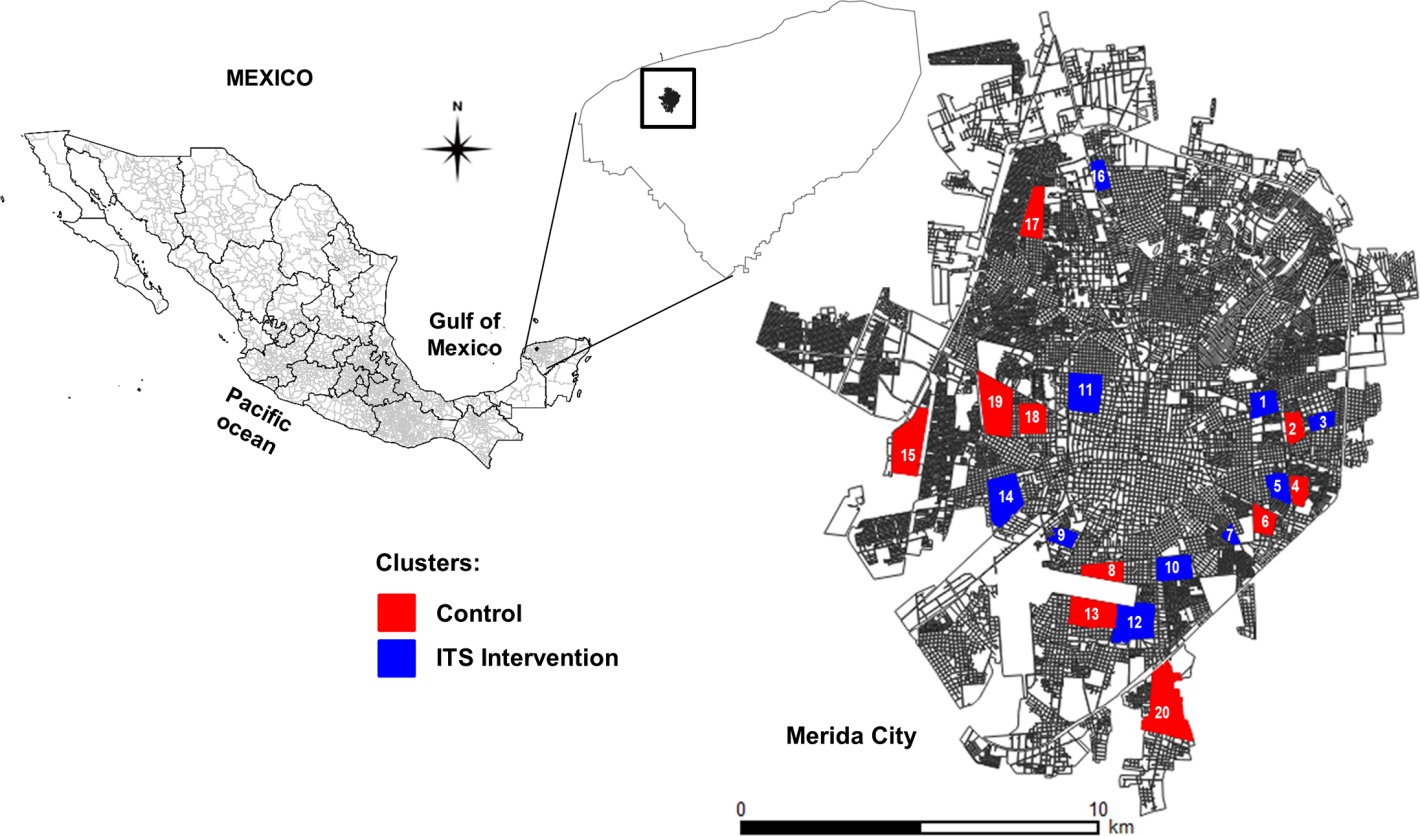
Location of treatment and control clusters within the city of Merida. The clusters with and without ITS are shown in blue and red, respectively. Source: INEGI, 2010 (http://www.beta.inegi.org.mx/app/biblioteca/ficha.html?upc=702825292805).

This study followed a core protocol developed by a consortium of researchers participating in a multinational project on eco-bio-social responses to dengue [45]. Briefly, a cluster-randomised design was applied to 20 geographic clusters (each cluster corresponded to a different neighbourhood) of 100 households each, with 10 clusters randomly assigned to either intervention or control arms of the study. In coordination with the local Ministry of Health, the study team selected the top 20 neighbourhoods in terms of their 2010–2012 cumulative dengue incidence (Fig 1). The randomization of treatments (ITS or not intervention) was then performed on these clusters (neighbourhoods). The randomization of treatments and paired design ensured a high consistency of key variables in intervention and control clusters. Once treatments were assigned, enrolment of households started in the most central block within the neighbourhood. All inhabited houses on the block were visited and their owners invited to participate in the project. Only houses that consented to participate in the intervention were included in the study. Household enrolment continued in neighbouring blocks until the target of 100 households per cluster was achieved. The average cluster size to reach 100 households was 14 city blocks (in Merida each city block has, on average, 25 houses). Not all premises within a block were enrolled in the study because they were either small businesses, empty, or householders were absent at the time of enrolment or declined to participate. Houses included in the study were typically single storey, made of cement-plastered blocks with a closed roof, and with no other ventilating features (i.e. ventilation bricks, eaves, etc.) other than windows.

### ITS intervention

**Fig 2:**
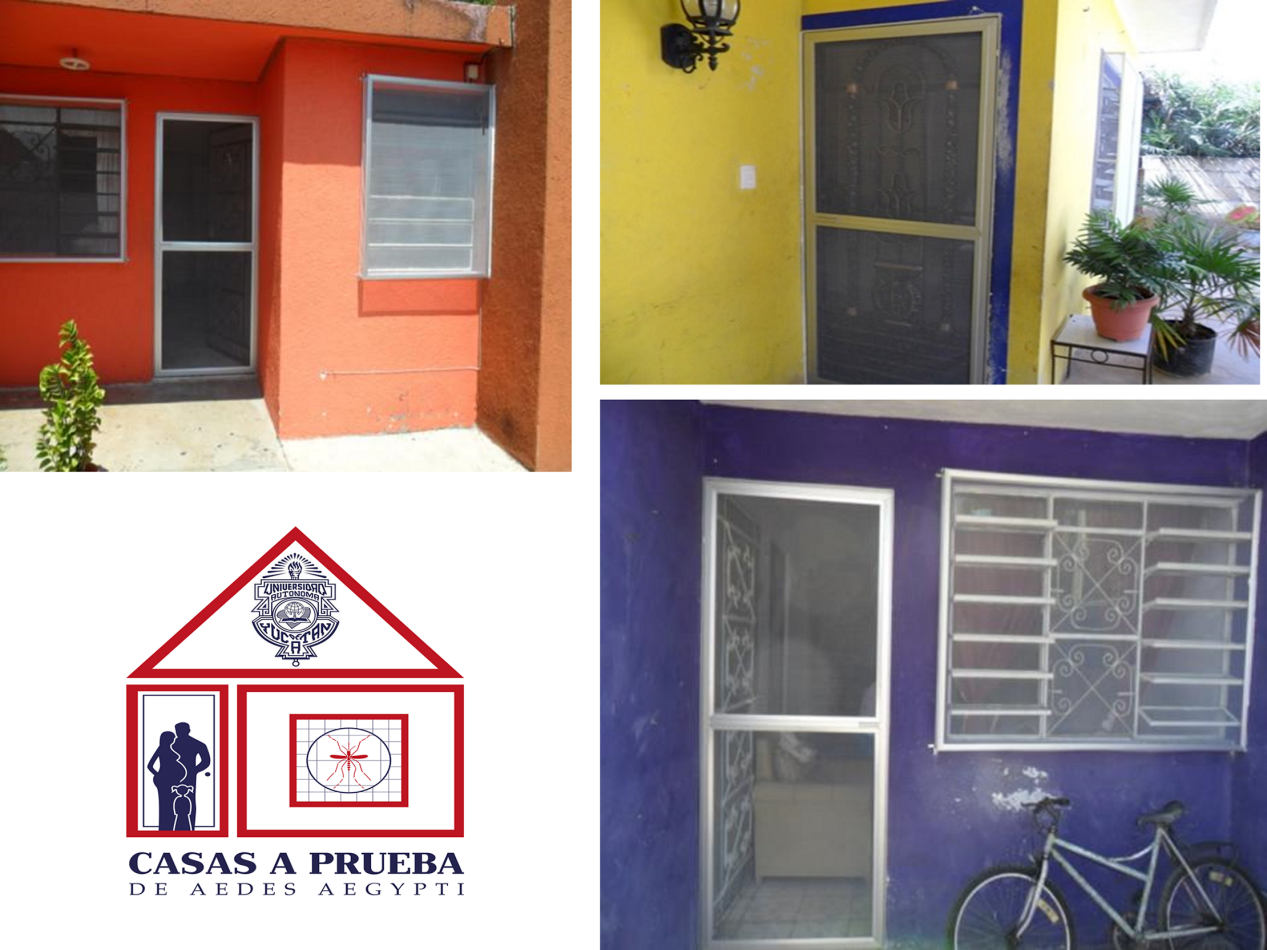
House screening with insecticide-treated netting. Photographs show housing improvement with insecticide-treated screens mounted on aluminium frames and fixed to external doors and windows of treated houses in Merida, Mexico.

As described in [34,35], Duranet LLIN material (0.55% w.w. alpha-cypermethrin-treated non-flammable polyethylene netting [145 denier; mesh1⁄4132 holes/sq. inch]; Clarke Mosquito Control, Roselle, IL, USA) was mounted in aluminium frames custom-fitted to doors and windows of houses in collaboration with a local small business. Houses were typically single storey flat roof house, made of cement plastered blocks with closed roof, without other ventilating areas than windows (Fig 2). Three teams of three persons/team installed ITS in an average of 100 houses per month (an average of 1.2 houses per day per team), and the installation took nine months to complete. A total of 844 households from intervention clusters (86% of coverage of houses which agreed to participate) were protected with ITS. An average (mean± standard deviation) of two doors (1.90±0.32) and four windows (4.40±0.84) by house were installed in each intervention cluster. During the installation, at least one person in every household received information from research staff about the proper use and maintenance of ITS. The total average cost of the ITS was US $91.5 per house.

As part of national policy in response to dengue outbreaks and entomological risk indices [46], routine vector control activities continued in all clusters throughout the study period. These activities included: outdoor spraying with organophosphates (chlorpyrifos-ethyl (2012–13) and malathion (2013–14)), indoor space spraying with a carbamate (propoxur, 2012–13) and a pyrethroid (deltamethrin, 2013–14) and larviciding with temephos.

### Intervention follow-up

Cross-sectional entomological surveys were conducted in intervention and control clusters as in [34,35]. Five cross-sectional entomological surveys were conducted: a baseline survey (September 2012) and four follow up surveys spaced approximately at 6-month intervals (March 2013, October 2013, March 2014, October 2014) post-intervention (PI) corresponding to wet, dry, wet, dry and wet seasons, respectively. Inside the clusters, the houses were randomized selected (n = 30/cluster). Briefly, indoor adult mosquito collections were performed in a randomly selected sub-sample of 30 houses from each cluster (total of 300 houses per arm). Indoor adult mosquitoes were collected with Prokopack aspirators [47] for a 15 min period per house. A standard procedure was implemented, starting the collections from the main enter of the houses and finishing in the rooms located at the back of the house. Collections within each cluster were performed on the same day between 09:00–15:00 hrs by 3 teams of 2 skilled collectors each. All mosquitoes collected were identified to species and sex.

No houses sampled in the control clusters had any existing window or door screens.

### Insecticide susceptibility status and kdr frequencies

The baseline study included insecticide susceptibility tests and genotyping for kdr. During the rainy season of 2012, a pool of *Ae*. *aegypti* eggs from each cluster were hatched from eggs collected from a network of weekly-serviced ovitraps placed in the clusters in both study arms. Batches of unfed 1–3 day-old females of the F1 generation/cluster were subjected to standard CDC bottle bioassays [48]. An additional 30 females/cluster hatched from ovitraps were stored at –70°C for molecular analysis.

The mosquito populations from each cluster were evaluated for resistance to two pyrethroids, permethrin, and alpha-cypermethrin (10 female adult mosquitoes per bottle were exposed; four replicates per test) using the suggested diagnostic doses (15 and 10 μg/mL respectively) and diagnostic time (knockdown at 30 minutes). Additionally, a control bottle test was set up in which mosquitoes were only exposed to bottle´s surface treated with acetone alone (without insecticide active ingredient). If knockdown between 3% and 10% was observed in the controls, the percent mortality was corrected using Abbott’s formula [49].

Genomic DNA was extracted from single whole mosquitoes or from a body part in a solution of 45 μl of H2O and 5 μl of Promega Taq DNA Polymerase10x Buffer with MgCl2 (Madison,116WI) in a 96 well PCR plate. Samples were incubated at 95°C in a BioRad icycler thermocycler for 15 minutes. The presence of kdr-1016I and -1534C alleles were assessed by real-time PCR reaction using the methodology described by [9].

The New Orleans and Rockefeller susceptible strains of *Ae*. *aegypti* were used as references for all CDC bottle tests. Genomic DNA from the Rockefeller strain was used as a susceptible (wild-type) control and DNA from previously genotyped individuals was used as positive controls for both kdr mutations. The CDC bottle tests and molecular assays were performed in the Entomological Bioassay Collaborative Unit (UCBE) of Yucatan University (Merida, Mexico) and CDC (Atlanta, USA) laboratories respectively.

### LLIN bioassays

Standard World Health Organization cone bioassays [50] were performed to determine the insecticidal activity of the ITS (LLIN material) at 6, 12, 18 and 24 months PI. On each survey date, net samples were obtained from three randomly selected houses in each intervention cluster (3 households x 10 clusters) and transported to the laboratory; the whole screen was cut immediately from centre (10 pieces of 25 cm x 25 cm per screen), wrapped in aluminium foil and then stored at 4 ^o^C for cone bioassays. As different nets had different deployment times, the age (time since installation) of nets distributed was recorded. New, unused Duranet material from the same batch used to make the ITS was also tested.

The WHO standard cone biossays were mostly implemented in ITS in good conditions (although during the study primarily the damaged screens were removed completely and replaced for new ones). ITS with holes were uncommon cases in the house sampled.

Groups of five non-blood fed, 1–3 day old *Ae*. *aegypti* from the New Orleans insecticide susceptible strain were exposed to each net sample for 3 minutes and then held for 24 hours in paper cups where they had access to a 10% sucrose solution. Post-exposure knock down (KD) was recorded at 30 minutes and 1 hour, and mortality was recorded after 24 hours.

Netting material was classified according to level of soiling (e.g., presence of dirt or other debris coating the white LLIN fabric) using a grey-colour palette (S1 Fig). The category for each net sample was defined in consensus among three different members of the team.

### Data analysis

The following indices were calculated for each sampling date: a) House positivity (presence of at least one) for adult female *Ae*. *aegypti*, b) House positivity for any *Ae*. *aegypti* adults, c) Number of female *Ae*. *aegypti* per house, and d) Number of total *Ae*. *aegypti* per house.

Logistic regression models (for binary variables) and negative binomial models (for count variables) were performed for each cross-sectional entomological evaluation survey as described in [34]. Odds ratios (OR) and incidence rate ratios (IRR) with 95% confidence intervals (CI) were assessed and significance expressed at the 5% level.

For WHO cone tests, the percent of KD at 60 minutes and mortality at 24 hours were calculated and corrected when the mortality in control replicates was >5 and <20% using Abbott's formula. To estimate the effect of soiling on the susceptible mosquito survival rate, a two way ANOVA test was performed: the measurement variable was the survival, and the two nominal variables were deployed time (at 6, 12, 18 and 24 months PI) and level of soiling (clean, soiled, very soiled and extremely soiled). Analyses were performed using STATA 12.0 (Stata Corp, College Station, TX).

Descriptive analyses of KD were obtained from different exposures to insecticides in CDC bottle bioassays, and classified according to following criteria [48]: 98–100% indicates susceptibility; 90–97% suggests resistance may be developing; less than 90% indicates resistance.

The frequencies of the 1016I and 1534C alleles were calculated using the following equation: [n heterozygotes +2(n homozygotes)]/2(total n mosquitoes analyzed)

### Ethics statement

This study received clearance from the ethical committee of the Ministry of Health of Yucatan. Written informed consent was obtained for each participating household (householder over the age of 18) in the beginning of the study.

## Results

### Impact of ITS on indoor *Ae*. *aegypti* adult mosquitoes

**Fig 3:**
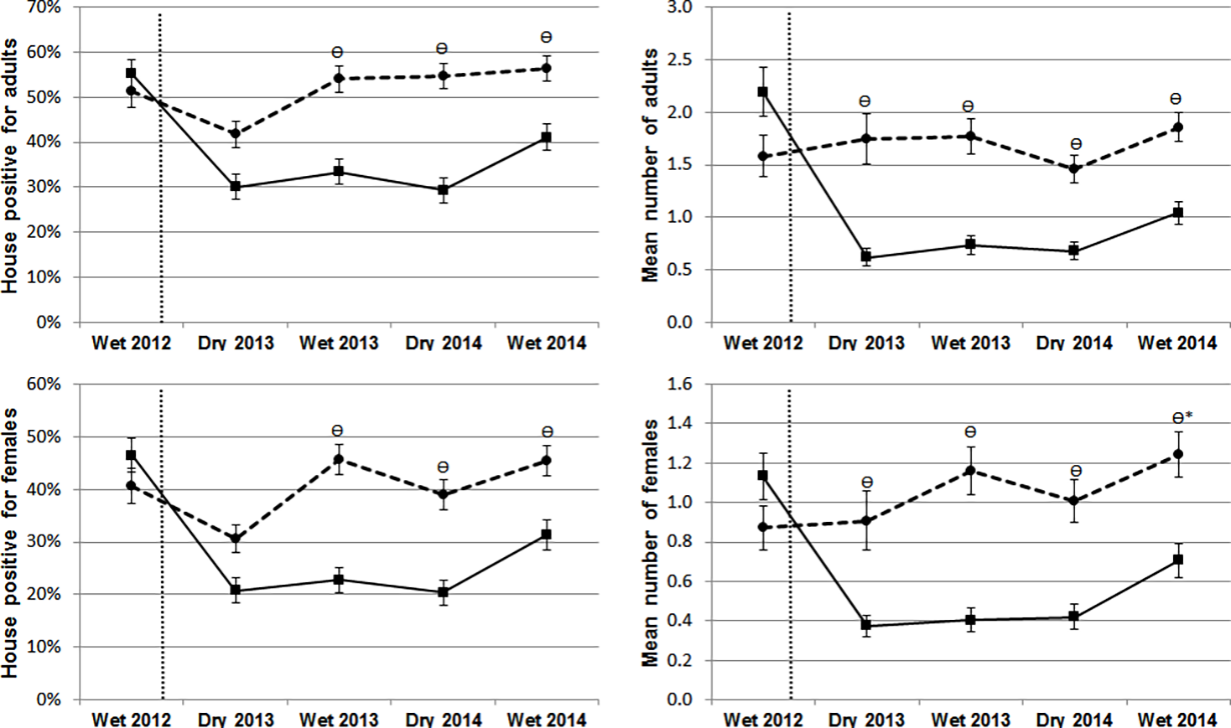
Impact of ITS on indoor *Aedes aegypti* adults. Comparison between treated (solid line) and untreated (broken line) clusters on the percentage of infested houses with *Ae*. *aegypti* (left) and their average abundance (right) in Merida, Yucatan, Mexico. The vertical dotted line represents the start of the intervention. The symbol ϴ denotes dates when the index was significantly different between ITS and control arms (with α = 0.05). Error bars show the standard error of the mean.

The indoor *Ae*. *aegypti* adult-based entomological indicators monitored for two years are shown in Fig 3. During the baseline survey, similar (non-statistically different) infestation levels were identified in both study arms. At the subsequent dry season survey (performed 6 months PI), no significant differences between intervention and control arms were observed regarding the presence of indoor adult females (OR = 0.59, 95% CI 0.28–1.27, P = 0.18) or adults (OR = 0.60, 95% CI 0.30–1.20, P = 0.15). However, in the ITS arm, significantly fewer houses were positive for both adult females (P = 0.004) and any adult *Ae*. *aegypti* during the following rainy season (12 months PI) and during the remaining entomological surveys through 24 months PI (Table 1).

**Table 1:** Results of logistic regression models (for presence-absence data) and negative binomial models (for count data) for adult indicators. Odds ratios (OR) and incidence rate ratios (IRR) with 95% confidence intervals are showed by entomological indicator for each cross-sectional entomological survey.

**Positivity**	**OR**	**P value**	**[95% Conf. Interval]**	
**Presence of adults**				
Rainy Season 2012*	1.17	0.671	0.56,	2.46
Dry Season 2013	0.60	0.149	0.30,	1.20
Rainy Season 2013	0.43	0.023	0.21,	0.89
Dry Season 2014	0.34	0.000	0.21,	0.56
Rainy Season 2014	0.54	0.009	0.34,	0.86
**Presence of females**				
Rainy Season 2012*	1.27	0.517	0.62,	2.59
Dry Season 2013	0.59	0.179	0.28,	1.27
Rainy Season 2013	0.35	0.004	0.17,	0.72
Dry Season 2014	0.40	0.001	0.23,	0.69
Rainy Season 2014	0.55	0.026	0.32,	0.93
**Abundance**	**IRR**	**P value**	**[95% Conf. Interval]**	
**Total adults**				
Rainy Season 2012*	1.38	0.285	0.76,	2.51
Dry Season 2013	0.36	0.011	0.16,	0.79
Rainy Season 2013	0.42	0.006	0.22,	0.77
Dry Season 2014	0.47	0.004	0.28,	0.78
Rainy Season 2014	0.56	0.017	0.35,	0.90
**Total females**				
Rainy Season 2012*	1.30	0.337	0.76,	2.23
Dry Season 2013	0.41	0.037	0.18,	0.95
Rainy Season 2013	0.35	0.003	0.18,	0.69
Dry Season 2014	0.42	0.011	0.21,	0.82
Rainy Season 2014	0.57	0.067	0.31,	1.04

*Baseline study

A significant reduction in the abundance of indoor *Ae*. *aegypti* adults and females was also observed in houses with ITS compared to control houses for the duration of the study (Fig 3, Table 1). At 6 months PI, total adult abundance was reduced on average by 64% in houses with ITS compared to control houses and this difference remained at approximately 50% for the remaining entomological surveys (range, 44–58%, Table 1). Female abundance followed the same trend, with an average reduction of 59% at 6 months PI and average reduction ranging between 43% and 65% during the subsequent surveys (Table 1).

### Bioefficacy of ITS under operational conditions

**Fig 4:**
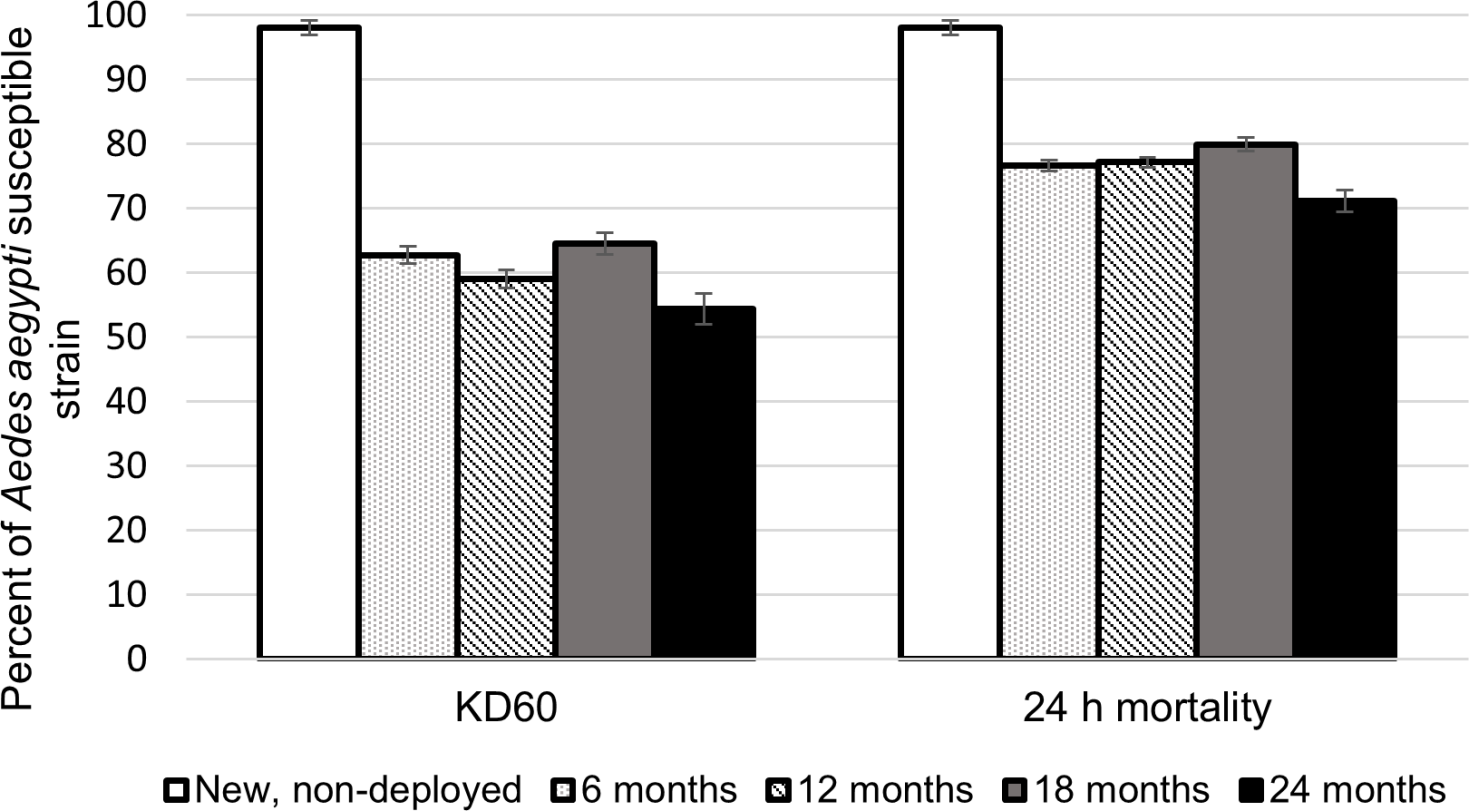
Cone bioassays performed at 6, 12, 18 and 24 months post ITS installation. Results of WHO cone bioassays after 3 min. exposure: knockdown at 60 minutes (KD60) and 24 h mortality and their standard errors of mean (SE) are shown for New Orleans susceptible *Ae*. *aegypti* strain.

The overall KD and mortality rate observed after WHO cone bioassays for new non-deployed nets assessed on the *Ae*. *aegypti* New Orleans susceptible strain was of 98% (SD±6.1 and ±5.3 respectively). Net samples collected at 6, 12, 18 and 24 months produced low KD levels (range 54–65%) at 60 minutes (Fig 4). Mortality at 24 hours were 71–80% at all time points (Fig 4).

**Fig 5:**
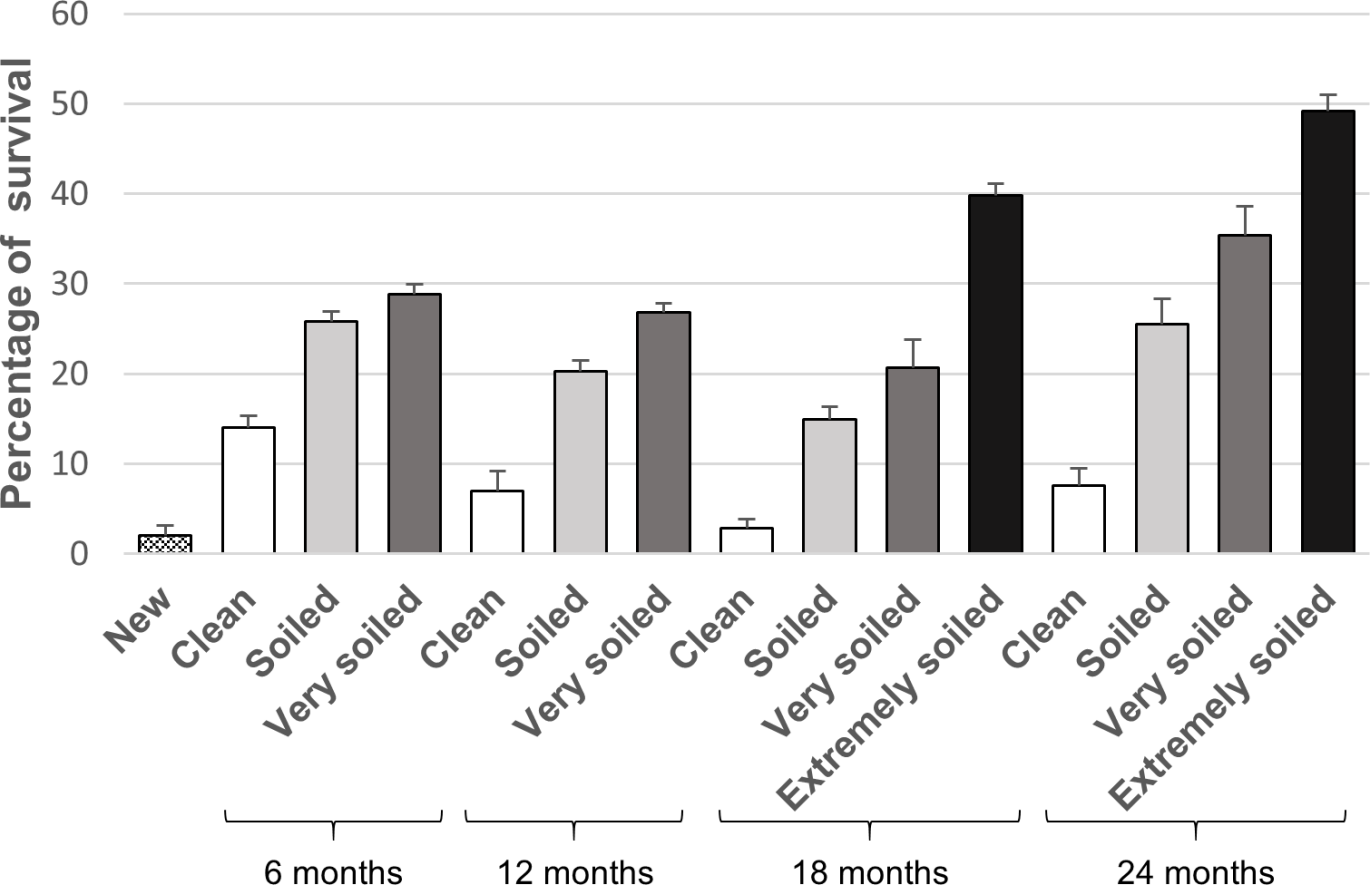
Survival in cone bioassays. Mean of percentage (±SEM) of susceptible *Ae*. *aegypti* survival after exposure to ITS with different deployed times and levels of soiling.

From the nets sampled at 6 months, the proportion of categories “clean”, “soiled” and “very soiled” LLIN was similar (30%, 33% and 37%, respectively). At 12 months, 58% of the total of nets sampled were categorized as ‘very soiled’. Extremely soiled LLINs were only obtained after 18 and 24 months PI, representing 30% of all net samples at those time points. The effect of time deployed and levels of soiling on mosquito survival were both significant (F_3_ = 30.2, P<0.001, and F_3_ = 231, P<0.001 respectively), but a significant interaction term was also observed (F_7_ = 2.2, P = 0.035). Significant differences in mean survival were observed only in 6 months deployed time when compared with the new-non deployed net (t = 4.5, P<0.001), and in the levels soiled (t = 6.8, P<0.001), very soiled (t = 9.6, P<0.001), and extremely soiled (t = 16.7, P<0,001). Significant interactions were observed between deployed time at 6 months and being very soiled (t = -3.8, P = 0.001), and at 18 months and being very soiled (t = -2.5, P = 0.012). Survival in the bioassay increasedas the nets became more soiled (Fig 5).

### Insecticide susceptibility status and kdr frequencies: Baseline study

High resistance to permethrin was observed for both intervention (%KD mean 38.2±4.9 S.E.) and control (%KD 39.1±4.6) clusters using the CDC bottle tests. However, houses from both study arms showed moderate resistance to alpha-cypermethrin (%KD for intervened clusters 94.1±1.8 and control clusters 90.9±2.7).

In mosquitoes genotyped for both kdr mutations (N = 295;n = 150 from the intervention arm and n = 145 from the control arm), the allelic frequency was higher for 1534C (0.93±0.12 and 0.88±0.09 for intervention and control arms, respectively) than 1016I (0.82±0.11 and 0.72±0.06 for the respective arms).

## Discussion

Results from this study illustrate the impact on *Ae*. *aegypti* that can be attained by using ITS. A house protected with ITS on doors and windows had at least a 50% less chance of having *Ae*. *aegypti* females in comparison with a non- screened house throughout the 2 year study period.

Other studies have evaluated the impact of interventions with LLIN on *Aedes* immature indicators, but not on indoor adult density. In Haiti, insecticide-treated bednets showed an immediate effect on immature based indicators, and extended for the following 5–12 months after their deployment [17]. In Thailand, ITC showed immediate effect on immature-based indicators at 6 months [23].

The primary entomological end-point for this trial was number of adult *Ae aegypti* inside houses. Our rationale for selecting adults was based on using a metric that is: a) most sensitive to the intervention, and b) most closely related to transmission risk. A previous study carried out in Acapulco, Mexico measuring adult *Ae aegypti* inside houses, reported similar protection levels during a first year of follow-up [35]. In Acapulco city, ITS achieved a protective effect for at least 600 days post installation for both adult- and immature-based indicators but, in this study a second treatment was implemented 14 months after the beginning of ITS intervention, based on targeted treatment of the most productive *Ae*. *aegypti* breeding sites [34, 35]. The LLIN protection conferred for at least 2 years was explained by the cumulative effect of the combined interventions. In the present study we observed an immediately effect of LLIN intervention on immature-based indicators, but was not consistently extended for more than 8 months. Sustainable interventions on larvae/pupae habitats can contribute to reducing the breeding sites and eventually the recruitment of individuals emerging from breeding sites [34, 35]. ITC interventions in combination with targeting productive breeding-sites in Mexico [16], Venezuela [16, 20] and Guatemala [19] have also indicated a synergistic effect on *Ae*. *aegypti* control.

The present study show evidence that ITS reduced significantly the indoor *Aedes* density for up to 2 years, despite the presence of resistance to pyrethroids in the local mosquito population. Insecticide resistance screening at baseline demonstrated that resistance was higher to permethrin than alpha-cypermethrin. High levels of permethrin resistance were detected in all clusters, but mosquito populations were moderately resistant or completely susceptible to alpha-cypermethrin. In both study arms, kdr frequencies were very high, close to 0.80 for 1016I and 0.90 for 1534C. Permethrin and alpha-cypermethrin were chosen for screening due to the historical use of pyrethroids and the current insecticide application strategy in the study sites. From 1998 to 2009, pyrethroids were the primary insecticides used for outdoor and indoor spraying to control adult mosquitoes, mainly using permethrin-based formulations. Alpha-cypermethrin was the active ingredient of the LLIN material used for the ITS intervention evaluated in this study.

Multiple factors could explain the lack of complete suppression of *Ae*. *aegypti* indoors by ITS. First, this method did not aim to reduce peridomestic breeding or outdoor abundance of mosquitoes. As intervention coverage on each block was not 100%, there is the possibility for mosquito breeding and human feeding even in the presence of screens. In Acapulco, Mexico, combining ITS with peridomestic larval control on the most productive larval habitats resulted in reductions of approximately 65% in abundance of indoor female *Ae*. *aegypti* [34], compared with the present study averaging a little more than 56% of reduction. This finding provides evidence of the importance of integrating ITS with additional methods. Secondly, some adults *Ae*. *aegypti* may not have contacted or survived contact with the ITS. Novel age-grading techniques [51] could provide more accurate estimates of entomological impact, particularly by quantifying the age structure of vectors inside houses with ITS versus unscreened controls. Social practices could also contribute to indoor presence of mosquitoes; *e*.*g*. it’s common in some neighbourhoods that householders keep the doors open for ventilation during the afternoon. In addition, doors of the houses protected with ITS were observed frequently to have been opened providing ease of entry of mosquitoes to the house.

A third explanation could be the loss of insecticidal power on the ITS and/or pyrethroid resistance in the local mosquito population. Exposure to sunlight, rain and dust can impact the insecticidal power of pyrethroid insecticides. Our study assessed the level of soiling as a factor affecting bioavailabiity and mosquito survival after exposure to LLIN. We found that most of the net samples (>60%) were soiled to at least some degree after two years and mosquitoes were more likely to survive as the level of soiling increased. This reduction of residual power is evidenced when comparing our results with other studies, using the same methodology, after exposing susceptible *Ae*. *aegypti* strains to deltamethrin-treated curtains (*e*.*g*.98-100% of residual insecticidal effectiveness after 12 months; [19,52]. Nevertheless, the field-efficacy of a LLIN may be underestimated if based only on standard cone bioassays [53]. Delayed mortality beyond the first 24 hours was not considered in this study, but it is known affect the survival of mosquitoes by reducing their life span [54]. Despite this, we observed that the insecticidal activity of ITS remained relatively high (>70% mortality), even after 2 years of use (the period considered in this study).

On the other hand it is unlikely that ITS, as used in this study, provided a protective effect by killing pyrethroid-resistant mosquitoes (it is more likely that they functioned simply as a physical barrier to prevent *Aedes* sp. from entering houses), in this case, one possibility is spraying alternative insecticides onto screens. Further studies should focus on the evaluation of the efficacy of re-impregnating ITS -which had partially lost its efficacy- as part of the resistance management strategies. As new insecticide-treated screening materials become available for mosquito control [55,56], it will be important to evaluate their inclusion within ITS programs that would benefit from greater residual potency at normal field conditions.

A few studies have related the development of insecticide resistance to the use of long-lasting insecticide treated materials. Temporal and spatial trends in Anopheles resistance (temporal increases in metabolic resistance and widespread distribution of kdr mutations) throughout eastern and western Africa [57] overlap with areas that have received long-lasting pyrethroid treated bednets [58]. Increases in kdr allele frequency have been linked to the increased coverage of insecticide treated bednets [59], which -in some cases- may have resulted in reduced impact of vector control interventions [60]. Nevertheless, it is difficult to confirm if this selection pressure is exerted mainly by the use of long-lasting insecticide treated materials, because additional selection pressures are likely involved, especially in urban contexts (e.g. selection pressures from insecticide sprays carried out by local ministries of health and the use of commercial aerosol sprays by householders). Although the results reported in this study may be explained in part by the reduced bioavailability of the insecticide active ingredient on the ITS surface over time, the physiological resistance of mosquitoes to the insecticide limiting the performance of the tool cannot be ruled out. Many of the studies relating pyrethroid resistance to the bio-efficacy of standard LLINs have been performed on malaria vector populations [61], with contradictory results [61, 62].

Improving housing can have significant protective effects against vector-borne diseases [63]. The present study demonstrated that LLIN material deployed as ITS can lower household infestations of *Ae*. *aegypti* for at least 2 years. As most human-mosquito contact with *Ae*. *aegypti* occurs indoors[64], the observed reduction in household *Ae*. *aegypti* infestation could impact virus transmission in a measurable way. Assessing the epidemiological impact of existing and new paradigms on *Aedes*-borne disease transmission remains a primary public health priority [1,2,15,65]. The body of entomological evidence, herein and previously, demonstrating the sustained impact and potential of ITS strongly supports the need for trials to quantify the intervention in reducing the burden of *Aedes*-borne diseases. Of particular interest is the evaluation of ITS within an Integrated Vector Management (IVM) scheme that includes additional complementary modes of vector control.

The level and duration of the protection against mosquitoes reported in this study can be compared with indoor residual spraying (IRS), historically the most effective long-lasting method for killing indoor-mosquitoes [66]. Although IRS has not traditionally been recommended for control of *Aedes* mosquitoes, when properly performed, it can have both an impact on *Ae*. *aegypti* infestation and dengue transmission [67, 68]. However, IRS is time consuming and expensive and requires regular retreatment. In terms of cost, IRS (using a carbamate) is US$16.5 per house for a single intervention (value estimated on actual cost of IRS applications for public health use in Mexico); so to protect a house during an entire year, IRS would need to occur four times (US$66/year), whereas the average cost of ITS for up to two years of protection was US $91.5 per house. The initial cost of ITS (or house screening) will be ultimately amortized considering the duration of the metal structures (which could last for more than 10 years) and one would only need to replace the mesh.

Controlling *Ae*. *aegypti* with ITS has advantages over many other approaches because they are permanently fitted on doors and windows, require little additional work or behavioural change by household members, and are associated with high overall satisfaction and acceptance levels [36]. Importantly, ITS also have the potential to provide long term household members protection from multiple other vector-borne diseases including malaria, lymphatic filariasis, and leishmaniasis in areas where they co-occur [10]. Ultimately, the suitability of ITS will depend on local construction characteristics, local acceptability of ITS, and local resources available for implementation. In conclusion, the results presented in this study further add to a growing body of evidence demonstrating that ITS is a promising new paradigm for *Ae*. *aegypti* control, even in areas where populations of this vector may be pyrethroid-resistant, and justify a second phase for larger trials (thousands of households) quantifying the effectiveness of ITS on epidemiological endpoints.

## Supporting information

S1 Fig Value scales for level of soiling. (TIF)
