# Supplementary figures and images for: House screening with insecticide-treated netting provides sustained reductions in domestic populations of Aedes aegypti in Merida, Mexico
Source: PLoS Negl Trop Dis. 2018 Mar 15;12(3):e0006283. doi: 10.1371/journal.pntd.0006283 (PMC5870999; doi:10.1371/journal.pntd.0006283)

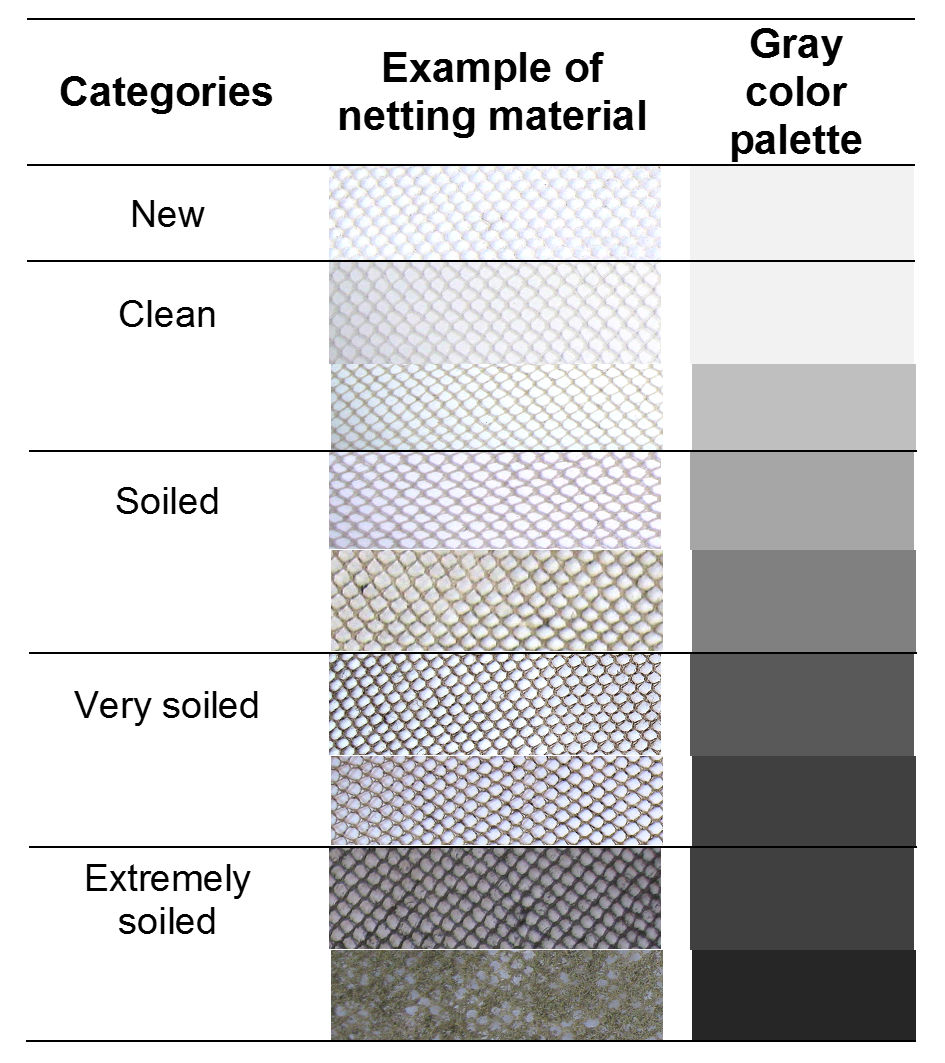

Supplement: S1 Fig — (TIF) [file pntd.0006283.s001.tif]
